# The Island Hospice model of palliative care

**DOI:** 10.3332/ecancer.2016.654

**Published:** 2016-07-07

**Authors:** Thembelihle Khumalo, Valerie Maasdorp

**Affiliations:** Island Hospice and Healthcare, 6 Natal Road, Belgravia, Harare, Zimbabwe

**Keywords:** Island Hospice, palliative care, cancer

## Abstract

There has been a substantial increase in cancer detections in Africa over years, and it has also been noted that higher number of individuals are affected by the later stages of cancer that lead to death. When it comes to cancer care, Zimbabwe is no exception with its ongoing palliative care related research, though still in its infancy. The need for advanced and more accessible palliative care to assist the vulnerable has been intensified by this increase in cancer prevalence. Island Hospice, which is a centre of excellence in palliative care has varying elements of the models that it employs to engage those most in need of palliative assistance, especially children and financially-challenged individuals.

## Introduction

Island Hospice and Healthcare (Island) provides palliative care services as per the World Health Organisation (WHO) model to all who are referred by clinic, hospital, doctor, or community structures. The WHO defines palliative care as an approach that improves the quality of life (QoL) of patients and families facing the problem of life-threatening illness. It is done through prevention and relief of suffering by means of early identification and impeccable assessment as well as treatment of pain and other problems such as physical, psycho-social, and spiritual [4]. Palliative care is relevant during the entire course of an illness and is seen to be vital to prevention, case-finding, testing, and adherence as well as care, support, and end of life care. As the first hospice in Africa (founded in 1979), Island is a centre of excellence in palliative care having trained other hospices in Africa. Through capacity building of community volunteers, health professionals, and palliative care specialists in other countries of the continent, Island has been emerging as a model of care that aims to provide quality care at a scale that counters the palliative care backlog in Africa, having arisen because of increasing prevalence of cancer and human immunodeficiency virus (HIV) cases. Sub Saharan Africa disproportionately bears the burden of HIV/AIDS in the world with 70% of people living with HIV [5].

## Background

Zimbabwe has an urgent and massive need for palliative care services as documented by the WHO global palliative care study that identified 1 in 60 Zimbabweans in need of palliative care, and the recent UNICEF/ICPCN study that identified a significant need for palliative care among children in all ten provinces of Zimbabwe [6]. [The Zimbabwe National Palliative Care Policy (August 2014) states: In a pilot project initiated by WHO in 2004 in five African countries which included Zimbabwe, it was estimated that a total of 208 600 people were dying from HIV and AIDS or cancer annually in Zimbabwe, of whom 200 000 people were dying from HIV and AIDS and 8 600 from cancer. The proportion of people needing palliative care was estimated at 1 in 60. Those dying from HIV and AIDS or cancer and suffering pain were estimated at 56 900. The report noted that the number actually needing palliative care was much higher because it should also include those suffering from serious illnesses but not dying, as well as those suffering from other diseases other than cancer or HIV and AIDS. In light of these considerations, WHO estimated that at least 1% of the population of any country would need palliative care.] According to the Zimbabwe National Cancer Prevention and Control Strategy (2014–2018), the country remains heavily burdened with HIV with an adult prevalence of 15%. The large number of people living with HIV results in an even higher number of people who will develop cancer in Zimbabwe. The same document asserts that the current cancer treatment and palliation services are unable to meet the existing demand.

This demand is compounded by the socioeconomic situation in the country which has resulted in social sectors, including health, all but collapsing. Zimbabwe, having been ranked in the bottom of the human development index (the HDI is a summary measure for assessing long-term progress in three basic dimensions of human development: a long and healthy life, access to knowledge and a decent standard of living) in the last five years [7], faces the pressures of rising unemployment as new labour act amendments resulted in over 20,000 [8, 9] people losing their jobs following a period where 711 firms in Harare closed down in 2013–2014. [The July 2013 National Social Security Authority (NSSA) Harare Regional Employer Closures and Registrations Report for the period July 2011 to July 2013 shows 711 companies in Harare closed down, rendering 8 336 individuals jobless.] Reduced health funding has resulted in the loss of experienced managers and professionals at all levels of the health system. The national health budget while having increased by 30 million [10–12] in 2016 is still three percent below the 15% proportion to national budget pledged by Zimbabwe in concert with the Abuja Declaration. Therefore, the common reality in Zimbabwe relating to a chronic illness diagnosis is profound economic crisis within a context where there is an ever increasing need for resources as well as primary healthcare service delivery.

With over 60 percent of the Zimbabwean population living in poverty [The Zimbabwe Statistical Agency (ZIMSTAT) Poverty, Income, Consumption and Expenditure Survey 2011–2012 report indicates that 62,6 percent of the country’s households are deemed poor; “*The analysis of poverty reveals that poverty is far worse in rural areas than in urban areas of Zimbabwe. 76 percent of the rural households are poor compared to 38,2 percent in urban areas, also an indication that 30,4 percent of rural people in Zimbabwe are “extremely poor” compared to only 5,6 percent in urban areas*.]; a large proportion of the chronically ill population are living in poverty and therefore lack the means to seek alternative routes for specialised or quality treatment outside of primary healthcare institutions. Many require cost effective healthcare services. Where those living in poverty are situated in remote areas, the need is for models of care that bring healthcare services closer to home, especially since symptoms and pain render the patient homebound and bedridden.

## Care, compassion, and impact

From inception Island adopted three key policies regarding the delivery of the care it set out to provide to the chronically ill: that this would be a non-fee-paying service; that it would be home-based (Island at that time, 1979, was facing a war-ravaged economy on the brink of change but plagued with similar resource constraints to what Zimbabwe faces today); and that a bereavement service—available to the community at large—would be an integral part of the service [13].

Inherent in this belief was the recognition that there was a need to provide access to better care for all people, with a life limiting disease, or those suffering bereavement. The model was inclusive of different medical disciplines [Island meeting records from 1979 show medical doctors, psychologists, social workers and pharmacists in attendance as founders mapped the goal and mission of Island. This tradition of employing interdisciplinary teams was eventually recognised in 2007 when Island received the Multi-disciplinary Team of the year award from IJPN (International Journal of Palliative Nursing)] to ensure the utmost medical professionalism, guaranteeing quality care for the patient, still being delivered in a cost effective way, which emphasised compassion in its approach by meeting the needs of the patient in the comfort of their own home. The goal was and remains to improve QoL for those facing the most difficult challenge of life-living with a life-limiting disease which often comes with chronic pain and symptoms.

Initially care was delivered to cancer patients and over time Island then began to expand its services and reach. 1987 saw the first AIDS patient on Island’s caseload. Two years later Island began bereavement workshops for children during the school holidays. By the late 90’s Island had transitioned to developing projects that delivered its programme of care in communities outside of its locale and funded by international partners. The principles remained the same; quality care, cost effective, compassionate, and always aiming to improve QoL.

As Island crossed over to the 2000s, there was a need to now extend the philosophy, knowledge, and skills inherent to the hospice model to provide access to better care for all people suffering from life-limiting diagnosis through training and capacity building. Two key principles led to this shift in focus; first—the need to scale up the access to care by capacity building communities in home-based care in order to own the delivery of palliative care to their constituents; second the need to engage health professionals within national governance structures in awareness as well as palliative care capacity building towards integration of palliative care as a vital component of the health delivery system.

Island was also given the opportunity through membership of the African Palliative Care Association to influence other hospices regionally. In the last 16 years, Island has trained and mentored palliative care professionals in Namibia, Zambia, Botswana, Kenya, and South Africa. Closer to home Island also made significant contributions to National Health policies and strategies. In 2003, Island assisted in the development of the National Ministry of Health and Child Welfare Community Home–Based Care Standards launched in June 2004. The standards are used as the guidelines in the provision of Home Based Care (HBC) services for HIV/acquired immune deficiency syndrome (AIDS) chronically and terminally ill patients. Island was also one of the key partners in the national protracted relief programme (PRP 2008–2011), specifically leading the Palliative Care Initiative (PCIZ) to improve the QoL of the most poor and vulnerable households faced with chronic illnesses. Island provided palliative care technical support to 11 major implementing partners and five of its own sub-partners through training and mentorship for home-based care volunteers and health professionals. In 2014, Island also assisted in the development and authoring of the National Palliative Care Policy August 2014. In the same year, the National Strategy for the Prevention and Control of Cancer 2014–2018 recognised Island not only as a pioneer of palliative care regionally, but also in providing multiple levels of in country training with a significant contribution to the emergence of community-based care as a health service delivery mechanism.

## The Island Hospice model of palliative care

The Island Hospice model of care delivers a holistic service to the patient and their family designed to meet their needs: physical, emotional, spiritual, social and cognitive. The holistic service is experienced in two ways. Firstly care is holistic in its trajectory; beginning from diagnosis of a patient, encompassing them and their families, and extending beyond end of life to bereavement follow-up for patient’s families. Secondly, the care delivered is holistic in its expertise as palliative care teams are interdisciplinary. The model therefore seeks to care for the whole of the individual (themselves and their primary connections/relationships that enhance their life) in a whole way (the necessary expertise in its diversity required to meet the multiple needs of the individual). The multidisciplinary team coverage of care thus includes medical aspects of the patients’ care and disease transmission, treatment adherence, psychosocial wellbeing, spiritual wellbeing, nutritional wellbeing of the patient, and family and social protection assessments and referrals. In essence, the Island model of palliative care is all about enhancing QoL and can therefore not only be applied to the treatment and care of life-limiting illness such as cancer and HIV, but in the treatment and management of chronic conditions such as diabetes and hypertension.

## Models of care

Over its almost four decades of service to the chronically ill across communities in Zimbabwe, Island has developed the following models for the delivery of care:

### 

#### 1. Clinic and hospital visits

Leveraging relationships built over time with health professionals in primary healthcare institutions as well as private medical facilities, referrals are made to Island. A team consisting of a palliative care nurse and social worker are dispatched to the patient, and pain and symptoms are assessed for follow-up care to be delivered when the patient is at home. As Island’s care services are founded on the WHO definition of palliative care which promotes holistic care, the patient is assessed from a physical and psycho-social perspective as well. Hospital visits are made based on referral from a physician, request from family member, or as part of a care plan protocol agreed regarding a patient already on the Island caseload.

#### 2. Home visits

Patients who are at home can also be visited by an Island team upon referral from a physician. The patient is assessed and care is delivered according to need. A homebound and bedridden client may require more than one visit a week in their care plan; a colon cancer patient may require a colostomy bag to be changed weekly etc. During a home visit the team also assesses the family and discusses possible interventions for strengthening the primary caregiver’s capacity to care for the patient while also coping with the burden of care. Children are particularly observed and engaged in assessment as they are the most vulnerable. A key aspect of the service that Island delivers is follow up bereavement care of patients’ families after the patient has passed on. The family is seen during follow-up bereavement visits and particular timings (first three months, six months after the death, one year after the death) are observed within the grief trajectory, although this is not formulaic in its application.

#### 3. Roadside clinics

Through the delivery of projects that deliver care to communities outside of Island locale, Island nurses have become recognised in communities where patients live remotely from primary healthcare institutions. As the Island model is non-fee paying, many other members of the community suffering ailments and chronic conditions would avail nurses as they make their rounds for home visits resulting in a roadside clinic. The Island nurse would stop the car and begin assessing patients on the side of the road. For many this became an easier and more accessible solution for accessing care than travelling the longer distance to healthcare facilities.

#### 4. Walk-in clinic services

The roadside clinic experience has helped us identify palliative care clinic sites in communities. The Island team sets a date monthly where those who require services are mobilised by Island volunteers to walk in and receive services. These also include paediatric palliative care clinics.

#### 5. Therapeutic and comprehensive bereavement care

Island offers a comprehensive bereavement service through its team of social workers and trained volunteer caregivers that provide or conduct:

- Individual counselling sessions
- Group therapy
  - children’s bereavement workshops and support groups
  - creative expression workshops for children
  - widows support groups

#### 6. Capacity building for caregivers

Island identifies primary caregivers with a particular focus on child-headed households, where young children and adolescents are caring for a chronically ill family member. These children are placed in young carers clubs where they have access to support from peers, are trained and watched by Island social workers and attend workshops on building coping mechanisms for the carers as well as learning how to provide adequate care from home. Primary caregivers who are not children are also provided with the same support mechanisms and opportunities for training. Over time Island has now developed a home-based care manual and a nutrition booklet that these groups leverage as tools to support the care they provide at home as well as the care they themselves need.

## How Island Hospice carries out its mandate to address the significant need for palliative care in Zimbabwe and Africa

Island’s response to the significant needs for palliative care employs a grassroots mechanism as well as engagement and relationship building with health governance structures.

Throughout various community based projects, the Island model of palliative care has provided a cost-effective quality healthcare service for a crosssection of vulnerable groups in Zimbabwean society. These projects identify volunteers within the community who are trained as Community Home Based Care Givers (CHBCs). These volunteers are trained by the team of specialised palliative care nurses and social workers in providing basic care and counselling to sick patients, their caregivers, and family. Their training curriculum includes follow-up refresher trainings by the Island team for quality assurance. Mobilising communities to take ownership of their care and service delivery to their most vulnerable constituents is the most sustainable way of meeting the significant need for palliative care in a resource constrained developing economy. Community volunteers have been a cost-effective way to redress the issues of access to care because of health worker shortages, distance to primary healthcare sites, and the economic burden associated with protracted healthcare over the period of a chronic illness. Furthermore, their training in palliative care provides them with the skills to provide quality healthcare in the home.

In regards to health governance structures, Island believes that working in conjunction with the Ministry of Health and Child Care (MoHCC) to empower government health workers with palliative care skills is a sustainable approach to changing the way communities address and care for terminally ill patients. This is because this is a key channel for integration of palliative care into the national health system. Capacity building strategies employed are culturally specific training curricula, interactive mentorship and supervision of health workers following training sessions, and focus on special populations in case management and referrals.

Training and capacity building therefore has been one of Island’s critical responses to improving access to palliative care in Zimbabwe. Currently Island is making strides in engaging as many sectors as possible; adding to and improving as well as creating awareness about the field of palliative and bereavement care in Zimbabwe. Key groups reached through these efforts are traditional healers, faith-based organisations, sects, rural communities, and non-governmental organisations.

## Impact

**Figure 1.**
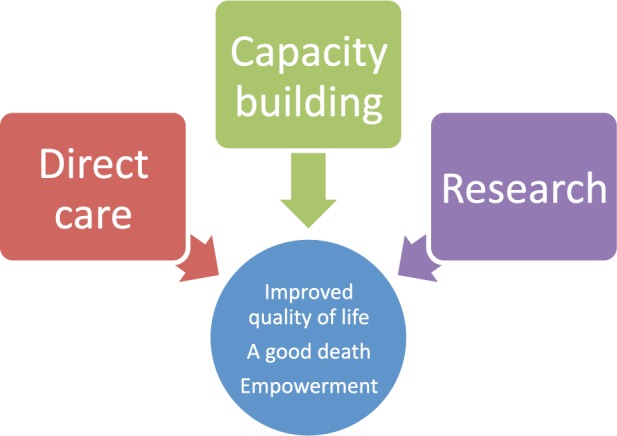
The Island Hospice theory of change (Island Hospice and Healthcare 2015).

Island’s goal to improve the QoL of those facing problems associated with life-threatening illness is always generally first achieved through the stories of change that patients share when they are receiving care (Figure 1). This is usually the experience that one has from reduction or relief of chronic pain and finally having a dignified, comfortable, and peaceful death, i.e. a peaceful end of life. Testimonials from stories of change are published on the Island website (www.islandhospice.care) and released periodically through a sponsored weekly radio programme.

However, QoL is also improved by improving quality of care. Therefore Island also measures its impact through the improved knowledge and skills within the community and civil society evidenced by increased referrals of patients to Island teams or staff and/or Island clinic sites in the community. This is one of the best ways to measure transfer of skill and knowledge to the community as it demonstrates an ability to identify patients as well as assess and refer cases appropriately.

Cancer survivors have given testimony of how Island has, through implementation of its programmes, helped in pain management and the overall wellbeing of these individuals by providing holistic palliative care support for them and their families. Where there is the inevitability of death, it is imperative to take into account the sensitivity of this burden on all who are involved.

## Conclusion

It is the aim of Island Hospice and Healthcare to bring about a positive change in the lives of the many individuals faced with the challenge of being affected by cancer-related terminal illness. This is carried out through various elements of research, capacity building, direct care, and the constant revision of outcomes of projects implemented by Island. The vision for the future is to provide equitable access to palliative care nationwide.

### Where does Island Hospice/health operate?

- Zimbabwe
- SADC

## Abbreviations

ARVs: Anti Retro-Virals
CHBCs: Community Home Based Care Givers
MoHCC: Ministry of Health and Child Care
MOU: Memoranda of Understanding
WHO: World Health Organisation
